# Caution about early intubation and mechanical ventilation in COVID-19

**DOI:** 10.1186/s13613-020-00692-6

**Published:** 2020-06-09

**Authors:** Martin J. Tobin, Franco Laghi, Amal Jubran

**Affiliations:** Division of Pulmonary and Critical Care Medicine, Hines Veterans Affairs Hospital and Loyola University of Chicago Stritch School of Medicine, Hines, IL 60141 USA

A fear of ventilator shortage with COVID-19 panicked politicians into demanding automakers to branch into ventilator manufacture.

Some experts have argued that mechanical ventilation should be employed early in order to prevent COVID-19 patients progressing from mild disease to more severe lung injury. This viewpoint has been expressed most forcefully by Marini and Gattinoni in a *JAMA Editorial* [1], where they attest that vigorous spontaneous inspiratory efforts can rapidly lead to patient self-induced lung injury (P-SILI).

P-SILI is thought to parallel ventilator-induced lung injury (VILI), an entity supported by decades of experimentation and randomized trials [2]. In contrast, P-SILI has surfaced only in the past 4–5 years [3]. Two research studies are commonly cited by authors warning about P-SILI [1, 3–5].

To induce hyperventilation, Mascheroni et al. [6] infused salicylate into the brainstem of spontaneously breathing sheep. The authors claim that the consequent ~ threefold increase in minute ventilation produced lung injury, and this was prevented by mechanical ventilation. Tidal volume (the focus of authors warning about P-SILI) [1, 3–5] increased from 178 to 235 ml. The proportional tidal volume in healthy humans would be 502 ml—much less than experienced by healthy pregnant women.

In a non-blinded, observational study, patients with acute respiratory failure who failed noninvasive ventilation had higher tidal volume than successfully managed patients. Carteaux et al. [7] concluded that high tidal volume predicted need for endotracheal intubation. Patients ultimately intubated were significantly sicker than non-intubated patients: more frequent immunosuppression (37.5% v 6.7%), higher SAPS II (41 v 30), and lower PaO_2_/FiO_2_ (122 v 177). Need for intubation was more likely precipitated by severity of underlying illness than tidal-volume size (which was found to be a marginal predictor). Tidal volumes in these two studies do not constitute a sound scientific basis for occurrence of P-SILI in patients with COVID-19.

Based on the P-SILI hypothesis, Gattinoni and coauthors advocate radical changes to ventilator management of patients with COVID-19. They claim that noninvasive options are of “questionable” value [5], “intubation should be prioritized”, [4] and delayed intubation will cause a P-SILI vortex that induces more severe ARDS [1].

They view heightened respiratory drive in COVID-19 patients as maladaptive, and recommend deliberate lowering of respiratory drive in these patients [1]. They claim that “near normal compliance … explains why some of the patients present without dyspnea” [5]. If a COVID-19 patient is severely hypoxic, normal lung compliance will not prevent dyspnea. Concurrently some COVID-19 patients are free of dyspnea despite substantial hypoxemia (dubbed “silent-happy hypoxia”) [8]. This arises because the level of hypoxemia per se is not sufficiently low to induce increased respiratory motor output and accompanying PaCO_2_ levels blunt the hypoxic response [2, 9].

To assess patient effort, Gattinoni and coauthors recommend inserting an esophageal balloon as a “crucial” step [5]. They specify that when esophageal-pressure swings increase above 15 cmH_2_O, “the risk of lung injury increases and therefore intubation should be performed as soon as possible” [5]. No experimental data exists to justify this assertion. Expressing vague and ill-defined concepts in mathematical terms gives them a specious air of respectability that cloaks lack of knowledge and perpetuates confusion. Equally important, manipulations of the upper airway while inserting an esophageal balloon in a dyspneic COVID-19 patient will escalate the risk for endotracheal intubation.

We are not recommending a desultory approach to instituting mechanical ventilation or saying that numbers are not important. When we learn that a patient is acutely and persistently hypoxemic despite supplemental oxygen, we immediately consider steps to institute assisted ventilation. But it is not possible to pick an oxygen saturation breakpoint at which the benefits of mechanical ventilation will decidedly outweigh its hazards across all patients [2]. To recommend instituting mechanical ventilation based on esophageal-pressure swings above 15 cmH_2_O [5] amounts to playing with fire.

Mechanical ventilation is lifesaving in severe respiratory failure, and few medical therapies equal its power [2]. While some COVID-19 patients can be managed with supplemental oxygen, patients with the most severe respiratory failure demand insertion of an endotracheal tube [8]. An endotracheal tube facilitates control over an unstable airway and enables precise regulation of oxygen, pressure and volume [10]. But the endotracheal tube brings in its wake a slew of complications [2]. Each day of mechanical ventilation exposes patients to complications and increases mortality [2].

Recommendations based on P-SILI for discontinuation of mechanical ventilation in COVID-19 patients are particularly radical. Marini and Gattinoni recommend that “weaning should be undertaken cautiously” [1]. Numerous studies demonstrate that physicians are unnecessarily cautious in assessing patients for weaning [2, 10]. To advocate “spontaneous trials only at the very end of the weaning process” [1] is a formula to increase mortality in COVID-19 patients—especially when an insufficient supply of ventilators is feared and some authorities recommend connecting four patients to a single ventilator.

The process of transforming thoughts about a new biological entity into material things (reification) takes years. Once existence of a new entity is corroborated through additional research, it acquires substance and is gradually accepted as approximating truth. History is replete with entities once viewed as real, now considered fiction (status lymphaticus, visceroptosis). At this time, the existence of P-SILI is based only on the shakiest of circumstantial evidence and has yet to be exposed to the acid-wash of experimental testing by differing scientists. Yet P-SILI is being promoted as a *raison d’etre* for a radical approach to mechanical ventilation in the time of the COVID-19 pandemic.

The true impact of mechanical ventilation in COVID-19 will never be known. It depends on whether intubated patients truly required mechanical ventilation or whether they could have been sustained with oxygen supplied by less drastic methods [8]. It is difficult to determine how many physicians have been influenced by P-SILI as a justification for preemptive mechanical ventilation as a preventive measure.

Even if high tidal volume and P-SILI play some role in the progression of respiratory failure in COVID-19 patients—for which there is no convincing evidence—this would not provide justification for liberal use of endotracheal intubation, for which there are decades of research documenting fatal complications.

## Data Availability

Not applicable.

## Abbreviations

VILI: Ventilator-induced lung injury
P-SILI: Patient self-induced lung injury
SAPS II: Simplified Acute Physiology Score II
